# Anticancer Effects of *Clausena hamandiana*: Ethanolic Extract Inhibits Cancer Cell Proliferation and Suppresses Lung Tumorigenesis in Mice

**DOI:** 10.3390/ijms27114743

**Published:** 2026-05-25

**Authors:** Chantana Boonyarat, Yoshihiro Hayakawa, Nutjakorn Samar, Nawinda Vanichakulthada, Rawiwun Kaewamatawong, Teeraporn Sadira Supapaan, Benjabhorn Sethabouppha, Pornthip Waiwut

**Affiliations:** 1 Faculty of Pharmaceutical Sciences, Khon Kaen University, Khon Kaen 40002, Thailand; chaboo@kku.ac.th; 2 Institute of Natural Medicine, University of Toyama, Toyama-shi 930-0194, Toyama, Japan; haya@inm.u-toyama.ac.jp; 3 Faculty of Pharmaceutical Sciences, Ubon Ratchathani University, Ubon Ratchathani 34190, Thailand; nutjakorn.sa.65@ubu.ac.th (N.S.); rawiwun.k@ubu.ac.th (R.K.); teeraporn.s@ubu.ac.th (T.S.S.); benjabhorn.s@ubu.ac.th (B.S.); 4 College of Medicine and Public Health, Ubon Ratchathani University, Ubon Ratchathani 34190, Thailand; nawinda.v@ubu.ac.th

**Keywords:** *Clausena harmandiana*, STAT3, apoptosis, anticancer activity, MAPK pathway

## Abstract

Cancer remains a leading cause of mortality worldwide, largely due to dysregulated apoptotic signaling and the persistent activation of oncogenic pathways. However, natural products are a promising source of multi-target anticancer agents. In this study, we investigated the anticancer activity and underlying mechanisms of *Clausena harmandiana* root extract and its major carbazole alkaloid, 7-methoxyheptaphylline, both in vitro and in vivo. High-Performance Liquid Chromatography (HPLC) chemical fingerprinting confirmed the presence of bioactive coumarins and carbazole alkaloids in the extract. Cytotoxicity assays demonstrated that the extract significantly reduced the viability of human colorectal adenocarcinoma (HT-29), human hepatocellular carcinoma (HepG2), human lung adenocarcinoma (A549–Luc2), and murine Lewis lung carcinoma (3LL–Luc2) cells in a dose- and time-dependent manner. Our mechanistic investigations revealed the activation of JNK signaling, downregulation of anti-apoptotic proteins (Bcl-2, Bcl-xL, and Mcl-1), and increased cleaved caspase-3 expression, indicating that mitochondrial apoptosis was induced. Notably, 7-methoxyheptaphylline markedly suppressed STAT3 phosphorylation in a concentration-dependent manner, comparable to the STAT3 inhibitor JSI-124. In a syngeneic 3LL–Luciferase2 lung cancer mouse model, oral administration of *C. harmandiana* capsules significantly reduced tumor growth and bioluminescence intensity compared with controls. These in vivo findings were consistent with the inhibition of STAT3 signaling and induction of apoptosis observed in vitro. Collectively, our results demonstrate that *C. harmandiana* exerts broad-spectrum anticancer activity through coordinated modulation of the JNK–STAT3 axis, leading to caspase-dependent apoptosis. These findings highlight its potential as a promising candidate for the development of STAT3-targeted anticancer therapies.

## 1. Introduction

Cancer is a multifactorial disease driven by genetic and epigenetic alterations that disrupt key regulatory pathways governing cell proliferation, survival, and apoptosis [1,2]. A hallmark of cancer progression is the dysregulation of apoptotic signaling, which allows malignant cells to evade programmed cell death, sustain uncontrolled growth, and acquire metastatic potential [3]. Importantly, failure of apoptosis is closely associated with therapeutic resistance and poor clinical outcomes, particularly in advanced-stage cancers [4].

Lung cancer remains one of the most lethal malignancies worldwide, accounting for a substantial proportion of cancer-related deaths [5]. Despite advances in molecularly targeted therapies and immunotherapy, lung cancer—especially non-small-cell lung cancer (NSCLC)—continues to exhibit low five-year survival rates due to late diagnosis, metastasis, and drug resistance [6]. In Thailand, lung cancer is the most prevalent cancer in males and remains a major public health concern [7]. These challenges underscore the urgent need for novel therapeutic strategies that can effectively restore apoptotic signaling in lung cancer cells.

Apoptosis is a tightly regulated cellular process essential for eliminating damaged or transformed cells [8]. It is mediated primarily through two interconnected pathways: the extrinsic death receptor-mediated pathway and the intrinsic mitochondrial pathway [9,10]. They converge on the activation of executioner caspases, particularly caspase-3, which orchestrate the biochemical and morphological features of apoptotic cell death [11].

The intrinsic apoptotic pathway is regulated by the Bcl-2 family of proteins, which control mitochondrial outer membrane permeabilization (MOMP) [12]. Upon apoptotic stimulation, pro-apoptotic proteins such as Bax and Bid promote mitochondrial dysfunction, leading to cytochrome c release and apoptosome formation, followed by caspase-9 and caspase-3 activation [13,14]. In lung cancer, the suppression of mitochondrial apoptosis and activation of caspases are common mechanisms underlying tumor survival and therapeutic resistance [15].

At the molecular level, the aberrant activation of oncogenic signaling pathways—including Janus kinase/signal transducer and activator of transcription 3 (JAK/STAT3) and JNK-plays a pivotal role in promoting the survival, proliferation, inflammation, and apoptotic resistance of cancer cells [16,17,18]. Persistent STAT3 activation has been widely reported in lung cancer and contributes to both chemo- and radioresistance by transcriptionally upregulating anti-apoptotic proteins, such as Bcl-2, Bcl-xL, and survivin, while suppressing caspase-dependent apoptosis [19,20]. Nuclear factor kappa-light-chain-enhancer of activated B cells (NF-κB) signaling is another key regulator of cancer cell survival and inflammation, frequently acting in concert with STAT3 to maintain a pro-tumorigenic microenvironment [21]. Constitutive NF-κB activation promotes the transcription of genes involved in cell proliferation, angiogenesis, metastasis, and resistance to apoptosis, including Bcl-2 family members and inhibitor of apoptosis proteins (IAPs) [22]. Crosstalk between the STAT3 and NF-κB signaling pathways has been shown to amplify anti-apoptotic signaling and contribute to aggressive cancer phenotypes [23]. Therefore, the simultaneous modulation of STAT3/NF-κB signaling and restoration of caspase-mediated apoptosis is a promising strategy for lung cancer therapy.

Natural products have historically served as a rich source of anticancer agents capable of targeting multiple signaling pathways with relatively low toxicity [24]. Several clinically important anticancer drugs, such as paclitaxel, vincristine, and camptothecin, exert their effects by modulating cell cycle progression and apoptosis [25,26,27]. These successes highlight the potential of plant-derived compounds to function as multi-target anticancer agents.

*Clausena harmandiana*, a medicinal plant in the Rutaceae family, has long been used in Thai traditional medicine for the treatment of inflammatory and gastrointestinal disorders [28,29]. Phytochemical investigations have identified carbazole alkaloids and coumarins as major bioactive constituents of *C. harmandiana*, including heptaphylline and 7-methoxyheptaphylline [24,30]. Previous studies have demonstrated that carbazole alkaloids isolated from *C. harmandiana* exhibit potent cytotoxic activity against several cancer cell lines, including lung cancer cells [31,32,33], which emerging evidence suggests is due to the modulation of apoptosis-related signaling pathways. Heptaphylline has been shown to induce apoptosis through Bid activation and suppression of Akt/NF-κB signaling, while 7-methoxyheptaphylline enhances death receptor 5 (DR5) expression via JNK signaling, thereby sensitizing cancer cells to Tumor Necrosis Factor (TNF)-Related Apoptosis-Inducing Ligand (TRAIL)-induced apoptosis [34]. However, the JAK/STAT3 pathway’s role in mediating the anticancer effects of *C. harmandiana*, particularly in lung cancer, remains largely unexplored.

Based on these observations, we hypothesized that *C. harmandiana* exerts its anticancer activity through coordinated modulation of apoptosis-related signaling pathways, specifically by restoring caspase-dependent apoptosis via suppression of STAT3 and JNK signaling. Therefore, in the present study, we aimed to investigate the antiproliferative effects of *C. harmandiana* ethanolic extracts on lung cancer cells in vitro and to evaluate the antitumor efficacy of its root powder in a mouse lung cancer model. We also sought to elucidate the underlying molecular mechanisms, with a particular focus on caspase activation and regulation of the STAT3 and JNK signaling pathways.

## 2. Results

### 2.1. HPLC Method Development and Chemical Fingerprint Analysis

**Figure 1:**
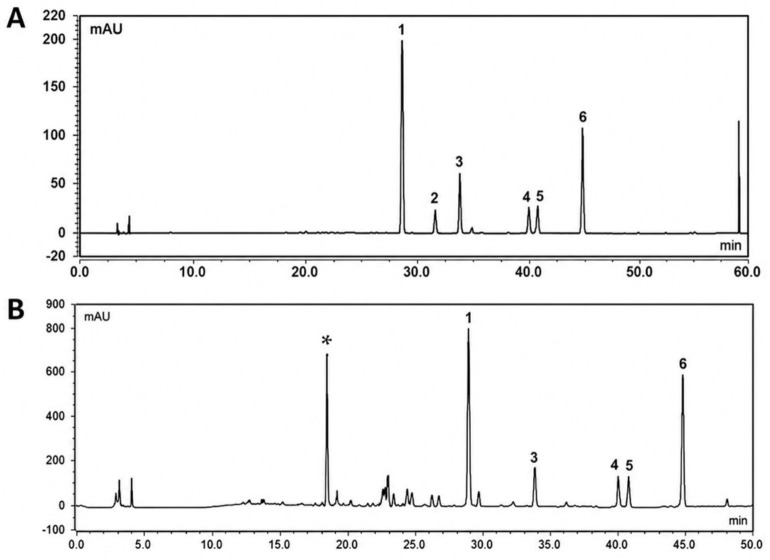
HPLC chromatographic analysis of *Clausena harmandiana* root extract. The chemical profile of the *Clausena harmandiana* root extract was analyzed using high-performance liquid chromatography (HPLC). (**A**) HPLC chromatograms of six reference standards: xanthoxyletin (1), osthol (2), nordentatin (3), 7-methoxyheptaphylline (4), heptaphylline (5), and dentatin (6). (**B**) HPLC chromatogram of the root extract showing the presence of clausine-K (*), xanthoxyletin (1), nordentatin (3), 7-methoxyheptaphylline (4), heptaphylline (5), and dentatin (6).

A qualitative and quantitative HPLC method was developed to perform a chemical fingerprint analysis of *C. harmandiana* root extract, able to clearly separate the six reference compounds with good resolution. The retention times of these standard compounds are shown in Figure 1A. Chemical fingerprint analysis of the *C. harmandiana* root extract was performed by comparing retention times and UV absorption spectra with those of authentic standards. The results demonstrated the presence of coumarin derivatives, including xanthoxyletin, nordentatin, and dentatin, but not osthol. Several alkaloids, namely clausine-K, heptaphylline, and 7-methoxyheptaphylline, were also identified in the extract. These findings are consistent with previous reports on the phytochemical constituents of *Clausena* species. Further comparative analysis of HPLC chromatograms obtained from different parts of the plant revealed distinct chemical profiles. Both the root bark and root wood extracts contained clausine-K, xanthoxyletin, nordentatin, dentatin, heptaphylline, and 7-methoxyheptaphylline. In contrast, none of these compounds were detected in the leaf extract. Representative HPLC chromatograms of extract from the root parts are shown in Figure 1B.

### 2.2. Cytotoxic Effects of Clausena harmandiana Extracts on HepG2 and HT-29 Cancer Cells

**Figure 2:**
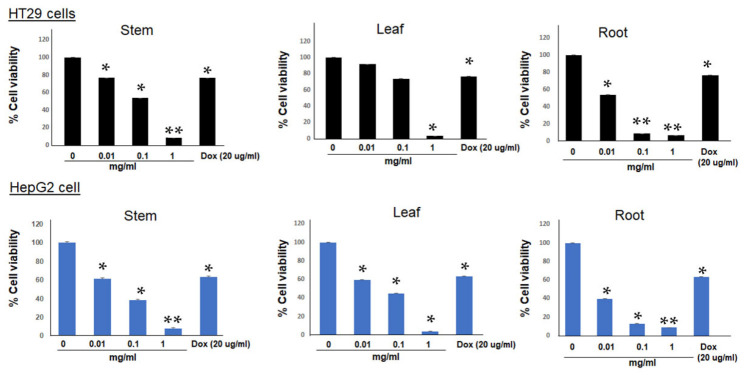
Effects of *Clausena harmandiana* stem, leaf, and root extracts on the viability of HT-29 and HepG2 cancer cells. Cells were treated with increasing concentrations of each extract (0, 0.01, 0.1, and 1 mg/mL) for 24 h, and cell viability was determined using the WST-8 assay. Doxorubicin (20 µg/mL) was used as a positive control. Data are presented as percentage cell viability relative to untreated control cells. Data are shown as mean ± SD from at least three independent experiments. (* *p* < 0.05, ** *p* < 0.01).

The cytotoxic effects of *Clausena harmandiana* extracts on cancer cell viability were evaluated in human hepatocellular carcinoma (HepG2) and colorectal adenocarcinoma (HT-29) cell lines. Cells were treated with extracts prepared from the leaves, stems, and roots of *Clausena harmandiana* at various concentrations, with doxorubicin used as a positive control. The results demonstrated that all tested extracts significantly reduced the viability of both HT-29 and HepG2 cells in a concentration-dependent manner. Of the different extracts tested, the root extract exhibited the strongest cytotoxic activity against both cancer cell lines, with IC_50_ values of 7.70 ± 0.002 µg/mL in HepG2 cells and 11.00 ± 0.002 µg/mL in HT-29 cells. The stem and leaf extracts also induced significant cancer cell death, although to a lesser extent compared with the root extract. In HepG2 cells, the IC_50_ values of the stem and leaf extracts were 58.00 ± 0.010 µg/mL and 59 ± 0.026 µg/mL, respectively, whereas in HT-29 cells, the IC_50_ values were 120.00 ± 0.020 µg/mL for the stem extract and 320.00 ± 0.016 µg/mL for the leaf extract. The dose–response relationships of the extracts with the HT-29 and HepG2 cells are shown in Figure 2.

### 2.3. Effects of Clausena harmandiana Root Extract on Apoptosis-Related Proteins in HepG2 Cells

To elucidate the molecular mechanisms underlying the *Clausena harmandiana* root extract’s anticancer activity, its effects on apoptosis-related signaling proteins were investigated in human HepG2 cells. Cells were treated with increasing concentrations of the extract, while N-acetyl cysteine (NAC) was used as a positive control. The results demonstrated that treatment with the *Clausena harmandiana* root extract markedly induced apoptotic cell death, as evidenced by a significant increase in the level of cleaved caspase-3 compared with untreated control cells.

**Figure 3:**
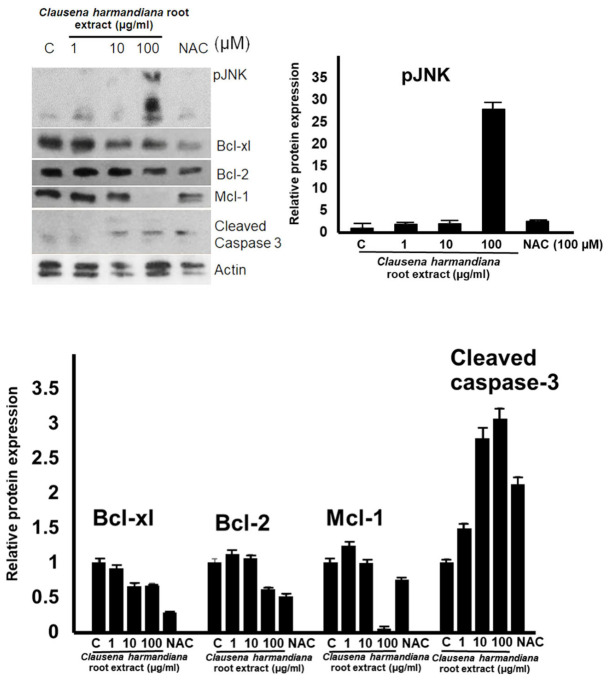
Effects of *Clausena harmandiana* root extract on JNK activation and apoptosis-related protein expression. Cells were treated with increasing concentrations of *C. harmandiana* root extract (1, 10, and 100 µg/mL) for 4 h. NAC (N-acetylcysteine) was used as an ROS scavenger control. Whole-cell lysates were subjected to Western blot analysis to determine the expression levels of phosphorylated JNK (p-JNK), anti-apoptotic proteins (Bcl-xL, Bcl-2, and Mcl-1), and cleaved caspase-3, with β-actin used as a loading control. Densitometric analysis of protein bands was performed and normalized to β-actin, and the results are presented as relative expression levels compared to the control group. Data are shown as mean ± SD from at least three independent experiments.

In parallel, the extract significantly suppressed the expression of key anti-apoptotic proteins, including Bcl-xL, Bcl-2, and Mcl-1, in a concentration-dependent manner. Furthermore, the extract enhanced the phosphorylation of c-Jun N-terminal kinase (JNK), a stress-activated protein kinase known to play a critical role in apoptosis regulation (Figure 3). This suggests that the JNK signaling pathway is involved in extract-induced apoptosis. Collectively, our findings indicate that the Song-Fa root extract promotes apoptotic cell death in HepG2 cells through activation of caspase-dependent apoptosis, downregulation of anti-apoptotic Bcl-2 family proteins, and activation of the JNK signaling pathway.

### 2.4. Cytotoxic Effects of Clausena harmandiana on Lung Cancer Cells

The cytotoxic effects of *Clausena harmandiana*-derived products were evaluated in lung cancer cell models using the WST-8 assay. The effects of both the isolated bioactive compound (7-methoxyheptaphylline) and the *Clausena harmandiana* extract on cell viability were assessed in murine lung carcinoma 3LL–Luc2 and human lung adenocarcinoma A549–Luc2 cells following 24 h of treatment.

**Figure 4:**
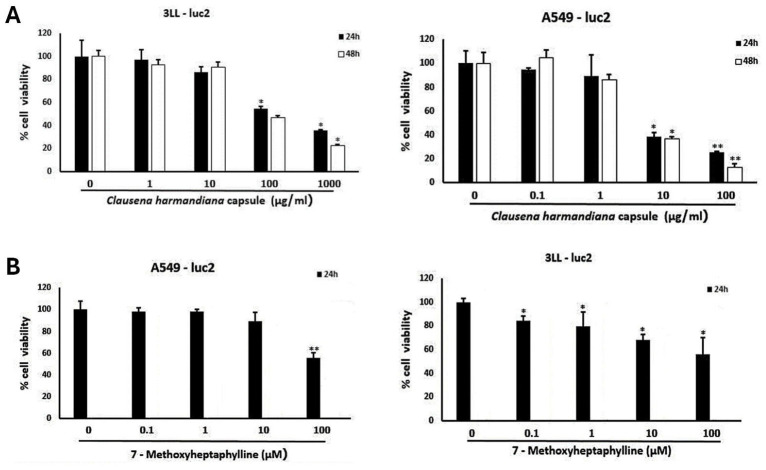
Effects of *Clausena harmandiana* extract and 7-methoxyheptaphylline on cell viability in lung cancer cells. (**A**) 3LL–luc2 and A549–luc2 cells were treated with increasing concentrations of *Clausena harmandiana* extract for 24 and 48 h. Cell viability was determined and expressed as a percentage of the untreated control. (**B**) A549–luc2 and 3LL–luc2 cells were treated with increasing concentrations of 7-methoxyheptaphylline for 24 h. Cell viability was expressed as a percentage of the untreated control. Data are presented as the mean ± standard deviation (SD) of three independent experiments. Statistical significance was determined relative to the untreated control (* *p* < 0.05, ** *p* < 0.01).

The results demonstrated that 7-methoxyheptaphylline significantly reduced the viability of both 3LL–Luc2 and A549–Luc2 cells in a concentration- and time-dependent manner. A more pronounced cytotoxic effect was consistently observed after 24 h of exposure, indicating sustained antiproliferative activity. Similarly, treatment with the *Clausena harmandiana* extract decreased the viability of 3LL–Luc2 cells, with enhanced cytotoxicity observed at higher concentrations and longer incubation times (Figure 4).

Collectively, these findings indicate that both the purified active compound and the formulated *Clausena harmandiana* extract possess significant cytotoxic activity against lung cancer cells. The consistent reduction in cell viability observed across different lung cancer models and treatment durations supports the potential of *Clausena harmandiana* extracts as promising candidates for further anticancer drug development.

### 2.5. 7-Methoxyheptaphylline Suppresses STAT3 Phosphorylation in A549 Lung Cancer Cells

**Figure 5:**
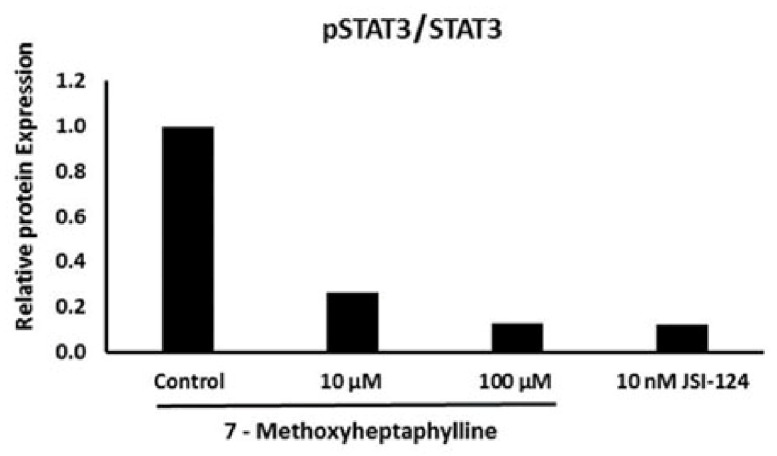
Effect of 7-methoxyheptaphylline on STAT3 activation. Relative protein expression of phosphorylated STAT3 normalized to total STAT3 (pSTAT3/STAT3) was analyzed following treatment of A549 lung cancer cells with 7-methoxyheptaphylline (10 and 100 µM). The STAT3 inhibitor JSI-124 (10 nM) was used as a positive control. Protein expression levels were quantified by densitometric analysis of Western blot bands and expressed relative to untreated control cells.

Treatment of A549 lung cancer cells with 7-methoxyheptaphylline significantly reduced STAT3 phosphorylation in a concentration-dependent manner. Notably, suppression at 100 µM was comparable to that observed with JSI-124, indicating that 7-methoxyheptaphylline effectively inhibited STAT3 activation (Figure 5). These findings suggest that modulation of the STAT3 signaling pathway may contribute to the antiproliferative and pro-apoptotic effects of the compound.

### 2.6. Oral Administration of Clausena harmandiana Capsules Suppresses Tumor Growth in Mouse Model

**Figure 6:**
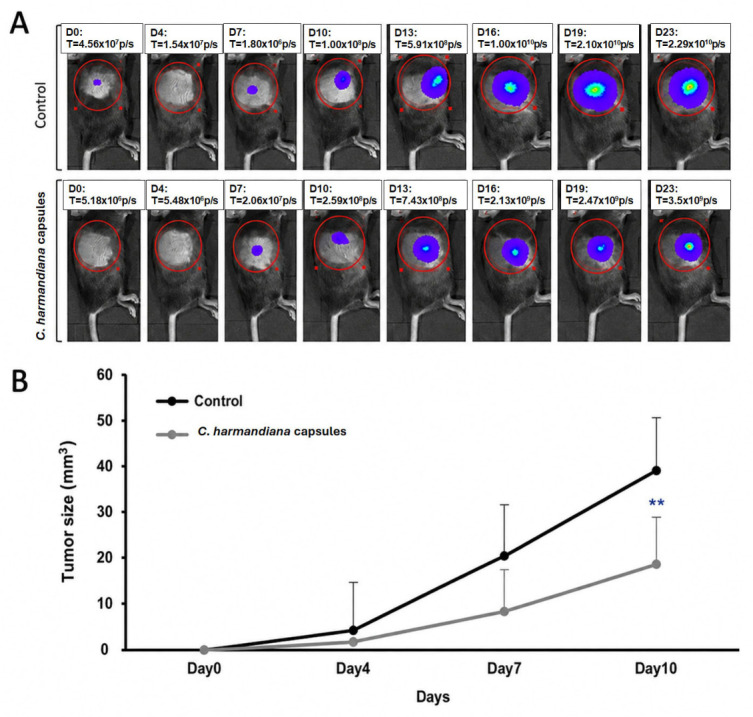
In vivo antitumor effect of *Clausena harmandiana* capsules in a 3LL–Luciferase2 lung cancer syngeneic mouse model. (**A**) Representative bioluminescence images of C57BL/6 mice subcutaneously implanted with 3LL–Luciferase2 cells and treated with either vehicle (control) or *C. harmandiana* capsules. Bioluminescent signals were acquired using an IVIS Lumina II imaging system. The red circles indicate the region of interest (ROI) defined over the tumor area for signal quantification. Photon flux (photons/sec) was quantified within the ROI using Living Image software and normalized to the background signal. A marked reduction in bioluminescence intensity was observed in the treatment group compared with the control group, indicating suppression of tumor progression. “D” represents the day of signal acquisition after tumor implantation, “T” represents the total photon flux measured within the ROI. Values expressed indicate the quantified bioluminescence intensity in photons per second (p/s). (**B**) Growth curves showing tumor volume (mm^3^) measured at the indicated time points (Days 0, 4, 7, and 10). Tumor volumes are presented as mean ± standard deviation (SD) (n = 8 mice per group). Statistical analysis was performed using one-way ANOVA followed by Tukey’s post hoc test to compare differences between groups. Mice treated with *C. harmandiana* capsules exhibited significantly reduced tumor growth compared with the control group. Statistical significance is indicated as (** *p* < 0.01), versus control.

To evaluate the antitumor efficacy of *Clausena harmandiana* capsules in vivo, C57BL/6 mice were subcutaneously implanted with 3LL–Luciferase2 lung cancer cells and monitored using bioluminescence imaging and tumor size measurement. Representative IVIS images demonstrated a progressive increase in photon emission intensity in the control group over time, indicating rapid tumor growth. In contrast, mice treated with *C. harmandiana* capsules exhibited markedly reduced bioluminescent signals. Quantitative ROI analysis confirmed that photon flux in the treatment group was consistently lower than in controls, suggesting effective in vivo suppression of tumor cell proliferation. Tumor size measurements further supported the imaging results. While both groups showed gradual tumor development after implantation, the control group exhibited a significantly steeper growth curve. By Day 7, tumor volume in the treatment group was significantly lower than that in the control group (* *p* ≤ 0.05). This difference became more pronounced by Day 10 (** *p* ≤ 0.01), indicating sustained inhibition of tumor progression following oral administration of the capsules. Overall, these findings demonstrate that *C. harmandiana* capsules effectively suppressed tumor growth in a syngeneic lung cancer model (Figure 6). The reductions in bioluminescence intensity and tumor volume suggest decreased tumor burden, which is consistent with the observed inhibition of STAT3 activation and induction of apoptosis in vitro. Collectively, the data support the potential of *C. harmandiana*-derived bioactive compounds as candidates for anticancer therapy.

## 3. Discussion

*Clausena harmandiana* has been utilized in traditional medicine and has undergone pharmacokinetic and toxicity evaluations in both animal models and humans, supporting its potential safety and translational relevance [35]. To further strengthen mechanistic interpretations and address the limitations associated with crude extract variability, the active constituent was isolated and identified through chromatographic separation. Of the isolated compounds, 7-methoxyheptaphylline was confirmed to be the major bioactive component responsible for the observed biological effects. The purified compound exhibited significant neuroprotective and anticancer effects comparable to or greater than those of the crude extract, thereby clearly demonstrating that the pharmacological effects of the extract are attributable to a defined single molecule rather than the crude extract as a whole. Previous studies have demonstrated that 7-methoxyheptaphylline exerts its biological effects through multiple signaling pathways, including Wnt/β-catenin, JNK-mediated DR5 expression, and TAK1 modulation, leading to cell cycle arrest and apoptosis in various cancer cell models. Collectively, these findings, together with reports on the bioactive constituents and pharmacological potential of *Clausena harmandiana*, provide strong evidence supporting 7-methoxyheptaphylline as a key active compound and highlight its promise for further therapeutic development [28,36,37,38,39,40,41,42,43,44].

Elevated intracellular reactive oxygen species (ROS), induced by cellular stress, therapeutic agents, or bioactive natural compounds, can function either as secondary signaling mediators or as potent oxidative stressors. Excessive ROS accumulation subsequently activates ROS-dependent c-Jun N-terminal kinase (JNK) signaling, thereby promoting apoptotic processes and suppressing cellular survival mechanisms. Furthermore, JNK activation has been reported to interfere with the oncogenic STAT3 signaling cascade, impairing its canonical phosphorylation, nuclear translocation, and transcriptional regulation of pro-survival and anti-apoptotic genes. The present study demonstrates that *Clausena harmandiana* root extract and its major bioactive compound, 7-methoxyheptaphylline, exert significant anticancer activity in both in vitro and in vivo lung cancer models. The data collectively indicate that the anticancer effect is mediated through modulating oxidative stress signaling, activating JNK signaling, suppressing STAT3 phosphorylation, and inducing mitochondria-dependent apoptosis. Notably, these findings are in line with previous reports on carbazole alkaloids isolated from *Clausena* species, which have been shown to exhibit cytotoxic and pro-apoptotic properties in various cancer models. STAT3 regulates the expression of numerous genes associated with the biological functions of both cancer and immune cells, making this signaling pathway an attractive therapeutic target. Inhibition of STAT3 has been reported to reverse immune suppression and restore protective anti-tumor and antiviral immune responses. Moreover, suppression of STAT3 signaling may alleviate its antagonistic interaction with NF-κB and STAT1 pathways, thereby promoting the release of Th1-type cytokines that are essential for host defense against malignancies and pathogenic infections. However, the present study further extends this knowledge by providing mechanistic evidence linking ROS generation to coordinated regulation of the JNK–STAT3 signaling axis [45,46,47,48,49].

In vitro cytotoxicity assays revealed dose- and time-dependent reductions in the viability of HT-29, HepG2, A549–Luc2, and 3LL–Luc2 cells following treatment with *C. harmandiana* extracts. Among the plant parts tested, the root extract exhibited the strongest antiproliferative activity, which is consistent with our HPLC profiling demonstrating higher enrichment of bioactive coumarins, including 7-methoxyheptaphylline. Previous phytochemical studies of *Clausena* species have similarly reported that carbazole alkaloids and coumarins are enriched in root tissues and contribute to their cytotoxic effects. The observed morphological alterations, such as cell shrinkage and membrane blebbing, further support apoptotic cell death and are consistent with earlier studies describing the apoptosis-inducing properties of carbazole derivatives.

Mechanistically, we observed increased phosphorylation of JNK, the downregulation of the anti-apoptotic proteins Bcl-2, Bcl-xL, and Mcl-1, and the activation of cleaved caspase-3, indicating the activation of the intrinsic (mitochondrial) apoptotic pathway. Similar activation of JNK signaling has been reported for several plant-derived carbazole alkaloids; however, direct evidence linking ROS generation to JNK activation in *Clausena harmandiana* has not previously been demonstrated. Importantly, pretreatment with N-acetylcysteine attenuated JNK phosphorylation, suggesting that ROS generation is an upstream event in extract-induced apoptosis. This observation is consistent with well-established ROS-mediated JNK activation mechanisms that promote mitochondrial dysfunction and apoptotic signaling in cancer cells [50,51].

Furthermore, 7-methoxyheptaphylline significantly suppressed STAT3 phosphorylation in a concentration-dependent manner. Constitutive activation of STAT3 is frequently observed in lung and colorectal cancers and is associated with enhanced tumor cell survival, proliferation, and immune evasion [52,53]. While previous studies on *Clausena*-derived compounds have primarily focused on cytotoxicity and apoptosis induction, research on STAT3’s potential as a molecular target remains scarce. Therefore, the inhibition of STAT3 signaling observed in this study demonstrates its novel contributions. This mechanism may explain the downregulation of Bcl-2 family proteins, known transcriptional targets of STAT3 [54], and thereby link STAT3 suppression to mitochondrial destabilization and caspase activation.

Importantly, our in vivo study using a 3LL–Luciferase2 syngeneic mouse model demonstrated that oral administration of *C. harmandiana* capsules significantly reduced tumor growth and bioluminescence intensity compared with controls. Previous in vivo investigations of *Clausena* species are relatively limited and have largely focused on crude extracts without detailed mechanistic validation. In this context, the concordance between the reduced STAT3 activation observed in vitro and the suppressed in vivo tumor progression strengthens the translational relevance of the ROS–JNK–STAT3 axis and highlights a mechanistically supported anticancer effect that extends beyond the descriptive observations reported in earlier studies.

Natural coumarins and carbazoles have been reported to exhibit anticancer activity by modulating oxidative stress and oncogenic signaling pathways [55]. Our findings both support these previous reports and expand upon them by identifying 7-methoxyheptaphylline as a potential STAT3-targeting phytochemical derived from *C. harmandiana.* Compared with previously studied coumarins and carbazole alkaloids, the dual modulation of ROS–JNK activation and STAT3 inhibition observed here suggests a broader and more integrated mechanism of action. Given the central role of STAT3 in cancer progression and therapy resistance, targeting this pathway with plant-derived compounds shows promise as a therapeutic strategy. In summary, we have identified 7-methoxyheptaphylline as a key bioactive compound responsible for the potent anticancer effects of *Clausena harmandiana.* Our findings demonstrate that both the *Clausena harmandiana* extract and its active constituent exert their activity through coordinated modulation of the ROS–JNK–STAT3 signaling axis, leading to mitochondrial apoptosis. Collectively, these results highlight the therapeutic potential of *Clausena*-derived compounds, particularly 7-methoxyheptaphylline, as promising candidates for further development in cancer treatment.

## 4. Materials and Methods

### 4.1. Preparation of Reference Standard Solutions

The reference standards dentatin, osthol, nordentatin, xanthoxyletin, heptaphylline, and 7-methoxyheptaphylline (Sigma-Aldrich, St. Louis, MO, USA) were individually dissolved in methanol (HPLC grade; Merck, Darmstadt, Germany) to obtain stock solutions at concentrations of 100 µg/mL each. Equal volumes of each standard solution were mixed to prepare a combined standard solution for chromatographic analysis.

### 4.2. Preparation of C. harmandiana Extracts

Root bark, root wood, and leaves of *C. harmandiana* were dried, powdered, and extracted separately. Each extract (10 g) was macerated with 100 mL ethanol (analytical grade; Merck, Darmstadt, Germany) at room temperature for 72 h, followed by filtration. The extraction process was repeated once under the same conditions. The combined filtrates were concentrated under reduced pressure using a rotary evaporator (Büchi, Flawil, Switzerland) to yield dried extracts. Extraction yields were calculated as percentage dry weight (% yield).

### 4.3. HPLC Conditions

HPLC analysis was carried out using a Dionex Ultimate 3000 HPLC system (Thermo Fisher Scientific, Waltham, MA, USA) equipped with a quaternary pump (LPG 3400), autosampler (WPS-3000), column oven, and photodiode array (PDA) detector. Chromatographic separation was achieved on a reversed-phase C18 column (Phenomenex, Torrance, CA, USA; 4.6 × 250 mm). The mobile phase consisted of 0.1% formic acid in water (Merck, Darmstadt, Germany) and acetonitrile (HPLC grade; Merck, Darmstadt, Germany), delivered at a flow rate of 0.8 mL/min. Detection was performed at a wavelength of 270 nm, and the total analysis time was 60 min. Data acquisition and processing were conducted using Chromeleon chromatography software (version 7.2) (Thermo Fisher Scientific, Waltham, MA, USA).

### 4.4. In Vitro Cytotoxicity Assay (WST-8 Assay)

The in vitro cytotoxic effects of the test compounds and plant-derived products were evaluated using a WST-8 colorimetric assay kit (Cell Counting Kit-8; Dojindo Molecular Technologies, Kumamoto, Japan). Human lung adenocarcinoma cells A549 (ATCC^®^ CCL-185™) stably expressing Luciferase2 (A549–Luc2), murine Lewis lung carcinoma cells (3LL–Luciferase2), human colorectal adenocarcinoma cells HT-29 (ATCC^®^ HTB-38™), and the human hepatocellular carcinoma cell line HepG2 (ATCC^®^ HB-8065™) were cultured in Dulbecco’s Modified Eagle Medium (DMEM; Gibco, Thermo Fisher Scientific, Waltham, MA, USA) supplemented with 10% fetal bovine serum (FBS; Gibco, Thermo Fisher Scientific, Waltham, MA, USA) and 1% penicillin–streptomycin (Gibco, Thermo Fisher Scientific, Waltham, MA, USA) at 37 °C in a humidified atmosphere containing 5% CO_2_. Cells were seeded into 96-well plates (Corning Inc., Corning, NY, USA) at a density of 1 × 10^4^ cells/mL (100 µL per well) and allowed to attach for 4 h. Cells were then treated with 7-methoxyheptaphylline or the capsules derived from *Clausena harmandiana* root at final concentrations of 0, 0.1, 1, 10, and 100 µM. Doxorubicin hydrochloride (Sigma-Aldrich, St. Louis, MO, USA) was used as a positive control, while untreated cells served as the negative control. For HT-29 cells, treatments were performed either with single agents or in combination with exosomes. After incubation for 24 and 48 h, 10 µL of WST-8 reagent was added to each well and incubated for an additional 1–2 h at 37 °C. Absorbance was measured at 450 nm using a microplate reader (BioTek Instruments, Winooski, VT, USA). All experiments were performed with at least three independent biological replicates, and each condition within an experiment was analyzed in triplicate wells (technical replicates). Data are presented as mean ± standard deviation (SD).

Cell viability was calculated using the following equation:

$$\mathrm{Cell}\;\mathrm{viability}\;(\%)=\frac{\mathrm{Absorbance}\;\mathrm{of}\;\mathrm{treated}\;\mathrm{cells}}{\mathrm{Absorbance}\;\mathrm{of}\;\mathrm{untreated}\;\mathrm{control}\;\mathrm{cells}}\times 100$$

### 4.5. Cell Lysate Preparation

To investigate the molecular mechanisms underlying the pro-apoptotic effects of the test compounds, cell lysates were prepared following treatment under the indicated experimental conditions. After incubation with the test samples, cells were harvested and lysed using an ice-cold lysis buffer containing 25 mM HEPES (pH 7.7), 0.3 mM MgCl_2_, 0.2 mM EDTA, 10% Triton X-100, 20 mM β-glycerophosphate, 1 mM sodium orthovanadate, 1 mM phenylmethylsulfonyl fluoride (PMSF), 1 mM dithiothreitol (DTT), 10 µg/mL aprotinin, and 10 µg/mL leupeptin (all reagents purchased from Sigma-Aldrich, St. Louis, MO, USA). Cell lysates were incubated on ice and subsequently centrifuged at 14,000 rpm for 10 min at 4 °C. The resulting supernatants were collected as total cell lysates and stored at −80 °C until further analysis. Protein concentrations were determined prior to immunoblotting.

### 4.6. Immunoblotting

Protein expression levels were analyzed by sodium dodecyl sulfate–polyacrylamide gel electrophoresis (SDS–PAGE) followed by immunoblotting. Equal amounts of protein from each cell lysate were mixed with loading buffer, denatured, and separated using SDS–PAGE. Proteins were then transferred onto Immobilon-P PVDF membranes (Millipore, Burlington, MA, USA) using a protein transfer system at 300 V for 1 h. Membranes were blocked with an appropriate blocking buffer to prevent nonspecific binding and subsequently washed with washing buffer. The membranes were then incubated overnight at 4 °C with primary antibodies against caspase-3, Mcl-1, survivin, Bcl-2, p-JNK, p-stat-3, Bcl-xL, and actin, and diluted in an antibody incubation buffer. After washing, membranes were incubated with horseradish peroxidase (HRP)-conjugated secondary antibodies for 1 h at room temperature. Immunoreactive protein bands were visualized using a chemiluminescent detection system, and protein expression levels were quantified by densitometric analysis relative to loading controls.

### 4.7. In Vivo Antitumor Evaluation Using 3LL–Luciferase2 Xenograft Model

The extract was encapsulated using commercially available empty hard gelatin capsules (450 mg/capsule). In brief, the dried extract was accurately weighed and inserted into the capsules using a manual filling device to ensure uniformity of content. No additional excipients were added. The filled capsules were then stored in a desiccator at room temperature until further use. Male C57BL/6 mice (6–8 weeks old) were randomly divided into two groups (n = 8 per group): a control group and a treatment group receiving the *Clausena harmandiana* capsules. Murine lung carcinoma 3LL–Luciferase2 (3LL–Luc2) cells (PerkinElmer, Waltham, MA, USA) were cultured under standard conditions in Dulbecco’s Modified Eagle’s Medium (DMEM; Gibco, Thermo Fisher Scientific, Waltham, MA, USA) supplemented with 10% fetal bovine serum (FBS; Gibco, Thermo Fisher Scientific, Waltham, MA, USA) and 1% penicillin–streptomycin (Gibco, Thermo Fisher Scientific, Waltham, MA, USA). Cells were prepared at a concentration of 1 × 10^5^ cells/mL in sterile phosphate-buffered saline (PBS; Gibco, Thermo Fisher Scientific, Waltham, MA, USA). After shaving the right lateral flank, mice were subcutaneously injected with 100 µL of the 3LL–Luc2 cell suspension. Twenty-four hours after tumor cell inoculation, mice in the treatment group were orally administered the *C. harmandiana* capsules at a dose of 500 mg/kg/day with a dosing volume of 200 µL/mouse/day for 10 consecutive days, while control mice received an equivalent volume of vehicle (0.5% carboxymethylcellulose sodium; Sigma-Aldrich, St. Louis, MO, USA). Tumor progression was monitored twice weekly, starting on Day 1 of treatment, by measuring tumor size using digital calipers (Mitutoyo, Kanagawa, Japan) and assessing bioluminescence signals using an IVIS Lumina II imaging system (PerkinElmer, Waltham, MA, USA). For imaging, D-luciferin potassium salt (PerkinElmer, Waltham, MA, USA) was administered intraperitoneally prior to image acquisition according to the manufacturer’s instructions. Bioluminescence intensity was quantified using Living Image software (version 4.2) (PerkinElmer, Waltham, MA, USA) and used as an indicator of tumor growth and tumor burden in vivo. All experimental procedures were approved by the Institutional Animal Care and Use Committee of University of Toyama and conducted in accordance with institutional and national guidelines (Approval No. A2022INM-5, A2025INM-08).

### 4.8. Statistical Analysis

Data were analyzed using IBM SPSS Statistics version 24 (IBM Corp., Armonk, NY, USA). Comparisons among multiple groups were performed using one-way analysis of variance (ANOVA), followed by Tukey’s post hoc test for multiple comparisons when a significant overall effect was detected. All data are presented as mean ± standard deviation (SD). A *p*-value of less than 0.05 was considered statistically significant.

## 5. Conclusions

In summary, *Clausena harmandiana* root extract and its bioactive compound 7-methoxyheptaphylline exhibited potent anticancer activity both in vitro and in vivo, involving ROS-mediated activation of JNK, suppression of STAT3 phosphorylation, downregulation of anti-apoptotic Bcl-2 family proteins, and induction of mitochondrial apoptosis. Oral administration of the capsules significantly suppressed tumor growth in a 3LL syngeneic mouse model. These findings highlight *C. harmandiana*’s status as a promising natural source of STAT3-targeting anticancer agents and support the further development of its bioactive constituents for cancer therapy. Therefore, the findings from this study may serve as a valuable foundation for future translational and clinical investigations in lung cancer patients. Further studies involving additional lung cancer models, tumor microenvironment analyses, and combination therapeutic strategies are warranted to validate and expand the clinical applicability of these findings. Collectively, this work provides a meaningful framework for the future development of novel anticancer agents derived from *Clausena harmandiana*.

## Data Availability

The original contributions presented in this study are included in the article. Further inquiries can be directed to the corresponding author.
